# Serum lactate monitoring may help to predict neurological function impairment caused by acute metabolism crisis

**DOI:** 10.1038/s41598-023-29506-y

**Published:** 2023-02-17

**Authors:** Dandan Liu, Qing Yao, Bin Song, Yue Sun, Hongyan Ju, Guanggang Li

**Affiliations:** 1 Department of Critical Care Medicine, The Seventh Medical Center of the General Hospital of the People’s Liberation Army of China, Beijing, China; 2 Department of Cardiac Surgery, Beijing Tiantan Hospital Affiliated to Capital Medical University, Beijing, China

**Keywords:** Experimental models of disease, Laboratory techniques and procedures, Predictive markers, Brain injuries, Neurological disorders, Outcomes research, Risk factors

## Abstract

To investigate the predictive value of serum lactate on neurological function impairment and the possible etiology. In this retrospective study, all the adult patients admitted to ICU more than 24 h after general anesthesia elective neurosurgery from January 2018 to January 2019 were recruited. The data of the serum lactate every 8 h during the 24 h of ICU admission were acquired and analyzed. 169 patients were included in the outcomes analysis. The average serum lactate after ICU admission was 3.7(3.4–4.1) mmol/L, higher than normal, and serum lactate elevated commonly after neurosurgery. The serum lactate at ICU admission (lactate_serum_0h) was not correlated with the outcomes, whereas the predictive value increased as the monitoring time was extended. The result indicated that lactate_serum_8h, the lactate_serum_16h, and the lactate_serum_24h were correlated with the primary outcome (difference of GCS scores before the surgery and after 24 h of ICU admission (ΔGCS_24h_) (*p* < 0.05). The lactate_serum_16h and the lactate_serum_ 24 h were correlated with all the outcomes except for the hospital LOS. The ROC curve suggested that the lactate_serum_24h achieved the best predictive value. Patients with serum lactate non-recovered trend after 24 h of ICU stay had decreased GCS scores and vice versa, as indicated by the graph of the dynamic changes in the serum lactate. The predictive value of the serum glucose/serum lactate ratio at ICU admission (G/L_serum)_ was analyzed, and the result indicated that it was correlated with the ΔGCS_24h_ (*p* < 0.05), the G/L_serum_ can predict neurological impairment earlier. Dynamic serum lactate monitoring and the G/L_serum_ at ICU admission have predict value on neurological function impairment after neurosurgery which might be attributed to ACMC.

## Introduction

The metabolism of the human brain is quite significant. It takes up 2 ~ 2.5% of the body weight while consuming 20% of the oxygen supply and 25% of the glucose supply of the whole body^1^. 60% of the energy utilization is employed for electrophysiological activities (e.g., the synaptic potential of glutamatergic neurons in gray matter), and the other 40% is for maintaining the homeostasis of the intracellular environment^2^. The brain has no energy reservation, its energy supply is primarily from the energy substrates in the circulation, and the energy substrates include glucose, lactate, ketone, glutamic acid, and glutamine which are different from other tissues and organs^1^.

Lactate has been investigated extensively and there is a famous hypothesis-Astrocyte-Neuron Lactate Shuttle (ANLS) proposed in 1994^3^. According to ANLS, the energy consumption of neurons increases with the activity of the neurons, and the Na + dependent transporter of glial cells and the Na^+^/K^+^ ATP enzyme are activated, the glial glycolysis and the extra-cellular lactate concentration increases, and lactate serves as the energy substrate. The utilization of glucose by neurons decreased with the increase of the excitability of the glutamatergic neurons, the utilization of lactate by neurons increased, and glycolysis of glial cells continued to supply lactate reserve. Existing studies have confirmed that neurons use lactate more effectively, even as the main energy substrate when glucose and lactate are available, since the increase of glycolysis can trigger oxidative stress injury and apoptosis, and glycolysis can decrease when lactate is the energy substrate^4,5^.

As an important cerebral metabolism substrate, the abnormal change of cerebral extra-cellular lactate concentration represents the imbalance of cerebral metabolism and can be used to predict neurological function impairment and outcomes. A study of 151 TBI patients by monitoring cerebral extra-cellular lactate and glucose indicated that a lower lactate/glucose ratio at ICU admission was correlated with ICU mortality and the lactate concentration on the first day of ICU admission was correlated with worse outcome (3-month GCS scores)^6^. If cerebral extra-cellular lactate concentration can be monitored earlier, the neurological impairment will be reduced and clinical outcomes can be improved with proper earlier treatment. However, an intra-cerebral micro-dialysis (CMD) catheter is required for monitoring the cerebral extra-cellular lactate concentration. CMD is an invasive operation and costs a lot, making it difficult to implement widely in clinical practice.

Existing research has confirmed the correlation between CSF lactate/glucose concentration; serum lactate/glucose concentration was optimal on the first day of ICU stay^6^, the serum lactate was proportional to the CSF lactate, the serum glucose was 1.67-fold to the CSF glucose^7^. The serum lactate can be tested by blood biochemical test or arterial blood gas analysis. The arterial blood gas analysis is quite simple, and the arterial gas analysis instrument is equipped in each ICU since it is the routine item of critical care patients’ monitoring. Moreover, the routine item of neurological patients’ monitoring conforms to the protocol of our ICU.

In this study, a hypothesis was proposed, i.e., serum lactate may be beneficial to predict neurological function impairment, and this hypothesis was tested. Existing research primarily investigated the correlation between serum lactate and other prognosis outcomes (e.g., mortality or tumor volume), whereas there has been rare correlation study between serum lactate and neurological function impairment. Moreover, the possible etiologies of neurological impairment were analyzed, especially the metabolic factor.

The Glasgow Coma Scale (GCS) has been employed for neurological function assessment for 40 years, and it has good accuracy, as indicated by its test result^8^. Thus, the GCS score was adopted for the neurological function impairment assessment.

## Methods

This was a retrospective study involving all the adult patients (≧18 years old) under general anesthesia elective neurosurgery with an ICU stay over 24 h after the surgery at The Seventh Medical Center of the General Hospital of the People's Liberation Army of China from January 2018 to January 2019. The study gained approval from the Institution Ethics Committee of The Seventh Medical Center of the General Hospital of the People's Liberation Army of China. Informed consent was obtained from all subjects or their legal guardians.

Following the standard protocol in our ICU, the serum lactate was monitored every 8 h during the first 24 h of ICU stay through arterial blood gas analysis. The following data from the patients’ medical records were obtained: demographics (sex and age), primary diagnosis, comorbidities, Acute Physiology and Chronic Health Evaluation (APACHE) II score; the serum lactate data every 8 h during the 24 h of ICU stay, the serum glucose data, mean arterial pressure (MBP), hemoglobin and partial pressure of oxygen at ICU admission, the difference of GCS scores before the surgery and after 24 h of ICU stay (ΔGCS_24h_), neurological complications during 24 h of ICU stay(delirium, agitation, hemorrhage, and infarction), fluid balance during the 24 h of ICU stay, hospital length of stay (hospital LOS), ICU length of stay (ICU LOS), 6-month Extended Glasgow Outcome Score (GOSE) (assessed through outpatient department follow-up or telephone follow-up), 28-day mortality, as well as in-hospital mortality.

Patients were excluded if they had diabetes, their ICU LOS was less than 24 h, or they had incomplete data. As shock status or other unstable circulatory conditions can cause hyper-lactatemia, patients with hypoxemia (the partial pressure of oxygen in arterial blood (PaO2) under barometric pressure without oxygen inhalation is lower than normal value ((100-age/3)mmHg), hypo-hemoglobin (hemoglobin lower than 130 g/l in males and 120 g/l in females (WHO criteria) or hypo-perfusion (pale, cold and wet limbs, oliguria (< 17 ml/h), skin motting score(SMS) > 0 or capillary refill time (CRT) > 3.5 s) were excluded when serum lactate was tested and collected. Additional exclusion criteria included non-elective neurosurgery patients as they might have unstable circulatory conditions.

The predictive value of the serum lactate to the following outcomes was explored, the primary endpoint was ΔGCS_24h,_ and the secondary endpoints were 28-day mortality, in-hospital mortality, neurological complications, 6-month GOSE, ICU LOS, as well as hospital LOS.

Note: The GCS scores assessments were conducted after the correction of physiologic abnormalities (severe hyper/hypothermia, severe hyper/hypoglycemia, severe acid–base imbalance, serve electrolyte imbalance, as well as endocrine abnormalities), which might affect the accuracy. The assessments were conducted after the cessation of analgesics and sedatives through the neurologic wake-up test (NWT)^9^.

### Statistical analysis

Statistical data description: Quantitative data are expressed as mean and standard deviation (M ± SD) (normal distribution quantitative data) and the median and 25th percentile–75th percentile (interquartile range) (M, P25-P75) (non-normally distributed data). Qualitative data were presented based on frequencies and percentages (%).

Correlation analysis: Multivariable logistic regression was employed for the risk factor analysis between ΔGCS_24h_ and continuous quantitative variables (PaO_2_, Hb, MBP, lactate_serum_ and the serum glucose/serum lactate ratio at ICU admission(G/L_serum_)). Binary logistic regression was adopted for calculating the correlation between serum lactate and binary variables (28-day mortality, in-hospital mortality, and neurological complications). Multivariable linear logistic regression was adopted to conduct the correlation analysis between serum lactate and other continuous quantitative variables. Ordinal logistic regression was employed to calculate the correlation between serum and grade variables (ΔGCS_24h_ and 6-month GOSE). A P value lower than 0.05 indicated that the difference achieved statistical significance.

### Ethical approval

The study was approved by the Institution Ethics Committee of The Seventh Medical Center of the General Hospital of the People's Liberation Army of China. Informed consent was obtained from all subjects or their legal guardians.All methods were carried out in accordance with relevant guidelines and regulations.

## Results

**Figure 1:**
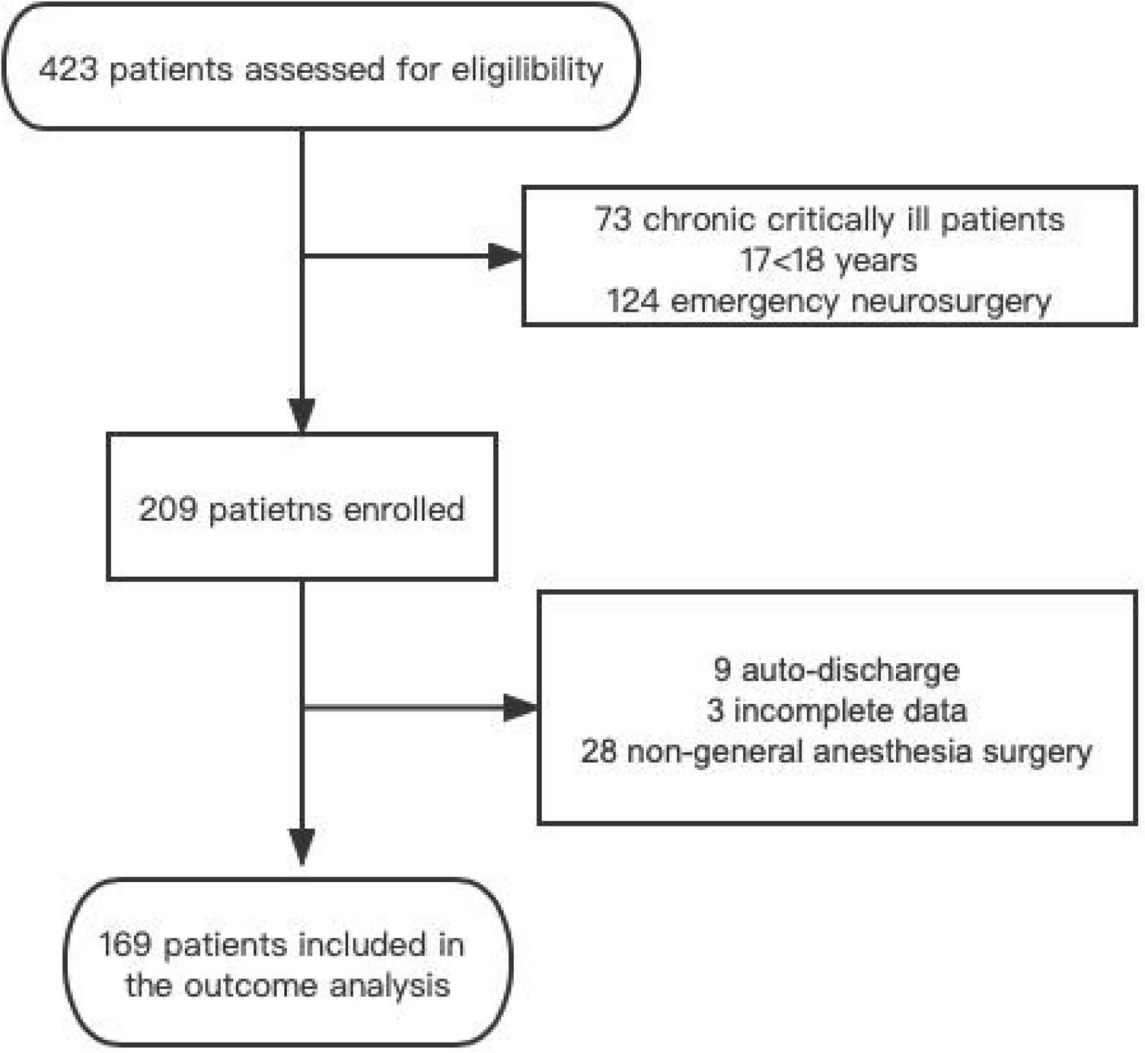
Flow of participants through the study.

423 patients were admitted to ICU during the study period, among which 209 patients were under general anesthesia elective neurological surgery. After 40 patients [incomplete data (n = 3), auto-discharge (n = 9), and non-general anesthesia operation (n = 28)] were excluded, the rest 169 patients were included in the analysis (Fig. 1).

As depicted in Table1, all the patients included in this study had a GCS score of 15 before the surgery, an APACHE II score of 9(9–10); the average age was 53.5 ± 0.9; 94 were male (55.6%); there were primarily intracranial tumor (102 tumor patients: 98 cases of glioma and 4 cases of other tumors) and intracranial aneurysm patients. Neurological complications were primarily delirium and agitation, with only 5 cases of intracranial hemorrhage and 1 case of small area infarction. It is noteworthy that smoking history might affect both central, cerebral, and peripheral perfusion and thus lactate production. According to the definition in the National Institutes of Health Stroke Scale, there were only 9 patients with a smoking history, such that the confounding influence of smoking history on the results was not considered.

**Table 1:** Characteristics of patients in the study.

Number of patients	169
Age	53.5 ± 0.9
Male sex	94 (55.6%)
Primary neurological diagnosis	
Tumor	102 (60.4%)
Glioma	98 (58%)
Others	4 (2.3%)
Intracranial aneurysm	39 (23.1%)
Others	28 (16.5%)
Comorbidities	
Hypertension	28 (16.6%)
Coronary heart disease	7 (4.1%)
Smoking history	9 (3.6%)
Neurological complications	
Delirium/agitation	104 (61.5%)
Hemorrhage	5 (3%)
Infarction	1 (0.6%)
At ICU admission	
GCS score	15
APACHE II score	9 (9–10)
Serum lactate	3.7 (3.4–4.1)
PaO_2_, mmHg	129 ± 34
Hb, g/L	135 ± 4
Capillary blood glucose, mmol/L	10.5 (8.7–12.9)
MBP, mmHg	81 ± 6
During ICU stay	
Lactate_serum_8h, mmol/L	3.5 (3–3.9)
Lactate_serum_16h, mmol/L	3 (2.7–3.6)
Lactate_serum_24h, mmol/L	2.2 (1.8–3.2)
Fluid balance, ml	219 (− 400–593)
GCS score	
GCS score decreased, n	48
GCS score non-decreased, n	121
Outcomes	
ICU LOS, day	1 (1–1)
Hospital LOS, day	15 (12–20)
MV time, min	160 (95–305)

Others: CAS (carotid artery stenting) + A-V M(Arteriovenous malformation) + epilepsy; PO2: partial pressure of oxygen; Hb: hemoglobin; ICU LOS: ICU length of stay; Hospital LOS: hospital length of stay; GOSE: extended Glasgow outcome scale.

PaO_2_ at ICU admission was 129 ± 34 mmHg, Hb was 135 ± 4 g/L and the MBP was 81 ± 6 mmHg. Binary logistic regression was adopted to conduct the independent factor analysis between ΔGCS_24h_ and continuous quantitative variables (PaO_2_, Hb, MBP, lactate_serum_ 0 h, and G/L_serum_), the results indicated that lactate_serum_ (*p* = 0.02, 95%CI 1.07 ~ 2.86) and G/L_serum_ (*p* = 0.007, 95%CI 1.2 ~ 3.14) were independently related to ΔGCS_24h._ PaO_2_, Hb, and MBP were not independently related to ΔGCS_24h._

The serum lactate at ICU admission (lactate_serum_ 0 h) was 3.7(3.4–4.1) mmol/L, above normal, it indicated serum lactate elevated commonly after surgery. The GCS scores after 24 h of ICU stay, 48 cases decreased and 121 did not decreased. 6 cases died within 28 days, and they were in-hospital mortality patients as well. The 6-month GOSE reached 8(8–8).

### Correlation analysis results between the serum lactate and the primary outcome and the secondary outcomes

**Figure 2:**
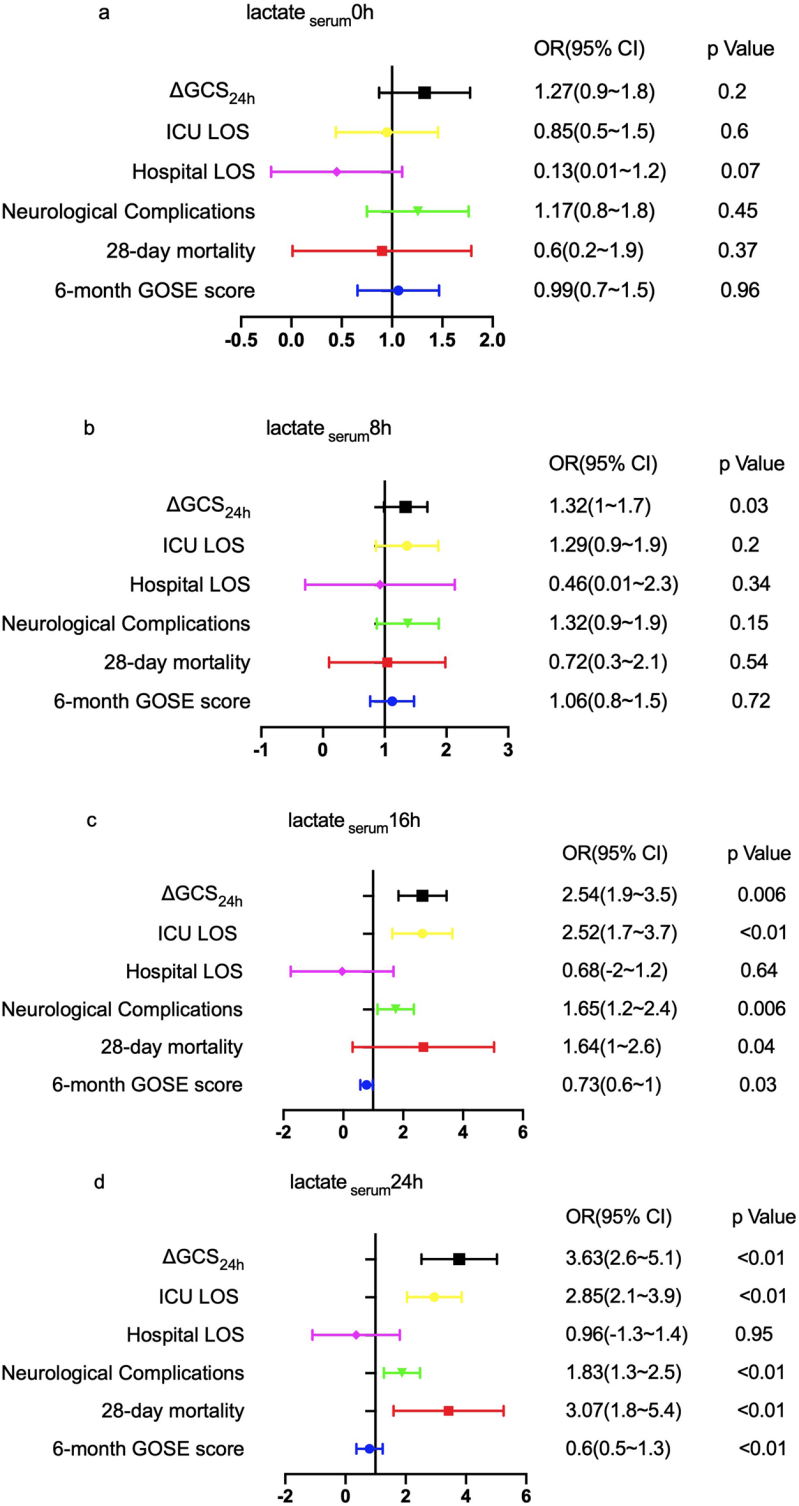
Correlation analysis of the serum lactate and the primary and secondary outcomes.

As depicted in Fig. 2, the lactate _serum_0h was not correlated with ΔGCS_24h_ (*p* = 0.2), ICU LOS (*p* = 0.58), Hospital LOS (*p* = 0.07), neurological complications (*p* = 0.45), 28-day mortality (*p* = 0.53) and 6-month GOSE (*p* = 0.96). In-hospital mortality was the same as 28-day mortality, the lactate _serum_0h was not correlated with in-hospital mortality too.

The predictive value of the serum lactate increased with the extension of monitored time. The serum lactate 8 h after ICU admission (lactate _serum_8h), the serum lactate 16 h after ICU admission (lactate _serum_16h), and the serum lactate 24 h after ICU admission (lactate _serum_24h) were correlated with ΔGCS_24h_ (*p* < 0.05). The lactate_serum_16h and the lactate _serum_24h were correlated with ICU LOS, neurological complications, 28-day mortality, and 6-month GOSE (*p* < 0.05), whereas they were not correlated with hospital LOS (*p* > 0.05).

**Figure 3:**
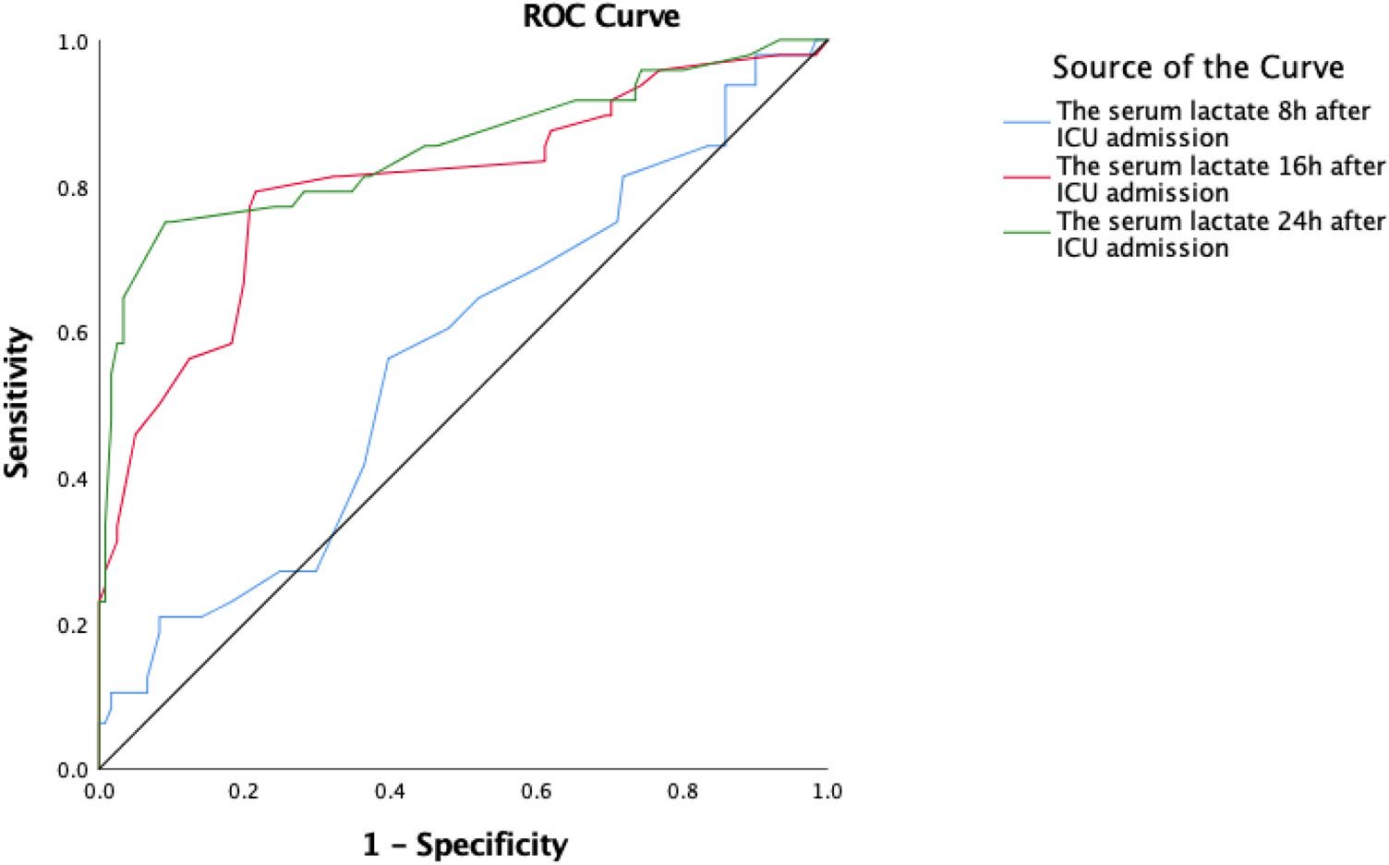
Receiver Operating Characteristic (ROC) Curve of the Serum Lactate to ΔGCS_24h_.

The receiver operating characteristic curve (ROC) was graphed between the lactate_serum_8h, the lactae_serum_16, the lactate_serum_24h and ΔGCS_24h_ after the correlation analysis. The results revealed that the predictive value of the serum lactate increased with the extent of monitored time, the predictive value of the lactate_serum_16h and the lactate_serum_24h was higher than the lactate_serum_8h, and the lactate_serum_24h was optimal. The AUROC of the lactate_serum_24h was 0.85, 95%CI 0.85 ~ 0.93, sensitivity 75%, specificity 90.9%, and the cut-off value reached 3.15 (Fig. 3).

**Figure 4:**
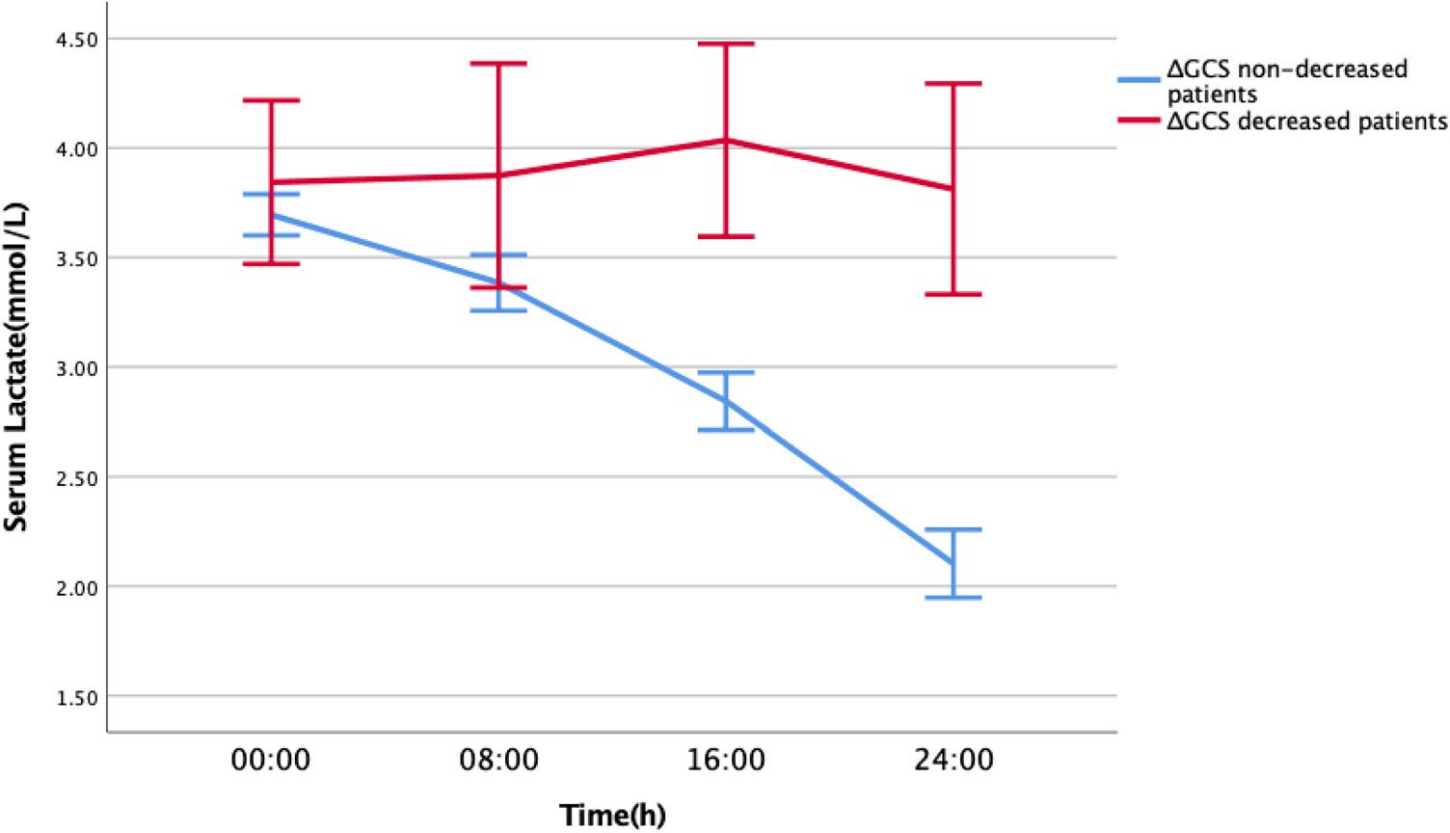
Dynamic changes of the serum lactate during 24 h of ICU stay after the surgery.

Dynamic changes in the serum lactate were graphed, it revealed that the patients with decreased serum lactate of the ΔGCS_24h_ did not recover during the 24 h of ICU stay, and patients with non-decreased serum lactate of the ΔGCS_24h_ tended to recover (Fig. 4).

### Correlation analysis results between serum lactate/serum glucose ratio (G/L_serum_) and outcomes

According to the previous studies, the correlation between the serum glucose/serum lactate ratio at ICU admission (G/L_serum_) and the outcomes was studied to find an index earlier to meet the clinical practice. As revealed by the results, the G/L_serum_ was correlated with ΔGCS_24h_ (OR 1.22, *p* = 0.008), 28-day mortality (OR 4.1, *p* = 0.001), 6-month GOSE (OR 0.74, *p* = 0.00) with a cut-off value of 2.4, was not correlated with neurological complications (*p* = 0.09) (Table 2). The G/L_serum_ had predictive value on neurological function impairment.

**Table 2:** Correlation analysis between the G/L_serum_ and the outcomes.

Variable	Outcomes	OR	95%CI	Cut-off value	*P*-Value
G/L _serum_ ratio	ΔGCS_24h_	1.22	0.02–0.1	2.4	0.008
	Complication	1.54	0.9–2.55	–	0.09
	28-day mortality	4.1	1.73–9.85	2.4	0.001
	6-month GOSE	0.74	− 0.2–0.07	2.4	0.00

G/L_serum:_ glucose/lactate; ΔGCS_24h_: difference of GCS scores before the surgery and after 24 h of ICU admission; OR:odd ratio.

## Discussion

Neurological function impairment can be caused by primary brain injury and secondary brain injury (caused by unstable circulatory or imbalance of cerebral metabolic homeostasis). Lactate elevation can be caused by the above factors too. In this study, patients with primary brain injury (acute brain injury, trauma, infection, and non-elective neurosurgery) and secondary brain injury (unstable circulatory) were excluded to minimize the confounding factors. Results of multivariable logistic regression revealed only lactate_serum_ and G/L_serum_ were independently related to ΔGCS_24h,_ PaO2, Hb, and MBP were not independently correlated with ΔGCS_24h_.

In the study, the result indicated that the serum lactate after the surgery was higher than normal (3.7 (3.4–4.1)) commonly. According to previous research, serum lactate was proportional to the CSF lactate^7^ and had the best correlation on the first day of ICU stay^6^, the elevated serum lactate can indicate the elevation of intracranial lactate concentration.

Though the lactate _serum_0h was above the normal level, it was not correlated with ΔGCS_24h_ and other outcomes, not consistent with existing research. A study of 102 gunshot patients found that serum lactate concentration was negatively correlated with the GCS scores at admission and positively correlated with in-hospital mortality^10^. A previous study of 213 moderate to severe TBI children revealed that the serum lactate concentration at admission was correlated with less mechanical ventilation time, shorter ICU LOS, hospital LOS, as well as higher mortality^11^. A study suggested that the serum lactate concentration during the operation was not correlated with all-cause mortality, whereas it was correlated with tumor volume^12^.

The differences from the existing studies may be explained below.

(1) *Different objectives* existing studies have largely focused on the predictive value of the serum lactate concentration on acute brain injury patients, whereas this study was focused on the predictive value of patients under general anesthesia elective neurosurgery; (2) *Different outcomes* tumor volume or all-cause mortality were analyzed in the existing studies, while we focused on the neurological function impairment 24 h after the surgery; (3) Different monitoring time, serum lactate concentration of glioblastoma surgery during operation in the previous study, we focused on serum lactate concentration after the tumor was removed when admitted to ICU.

In addition, we found the predictive value of the serum lactate increased with the extent of monitoring time, the lactate_serum_16h and the lactate _serum_24h were correlated with ΔGCS_24h_, ICU LOS, neurological complications, 28-day mortality, and 6-month GOSE (*p* < 0.05). The predictive value of the lactate_serum_24h on ΔGCS_24h_ was the best, the AUROC of the lactate_serum_24h on ΔGCS_24h_ was 0.85, 95%CI 0.85 ~ 0.93, sensitivity 75%, specificity 90.9%, cut-off value 3.15. Graph of the dynamic changes in the serum lactate concentration indicated that the serum lactate of the ΔGCS_24h_ decreased patients had no recovered trend during the 24 h of ICU stay, and the serum lactate of the ΔGCS_24h_ non-decreased patients had a recovered trend (Fig. 4).

To find an index indicating neurological function impairment earlier, we investigated the predictive value of the G/L_serum_ ratio according to the existing studies. The results indicated the G/L_serum_ was correlated with ΔGCS_24h_, the G/L_serum_ had predictive value on neurological function impairment.

It was the same or similar to the existing studies. Previous research found the concentration of CSF lactate/ pyruvate and glutamate increased and the CSF glucose decreased in the abnormal cerebral metabolism patients by monitoring CSF lactate and glucose concentrations of TBI patients with CMD^13^. In a previous study, 89 cases of moderate to severe TBI patients were monitored for 72 h continuously, the result suggested that 76% had decreased cranial extra-cellular glucose concentration in 84 patients with adequate fluid resuscitation, 93% had increased lactate/ pyruvate concentration, and 74% had cerebral metabolism crisis^14^. In a previous study, 10 out of hospital cardiac arrest patients who underwent hypothermia were investigated, and their CSF lactate and glucose concentrations were monitored hourly by CMD. The result found that the lactate/ pyruvate concentration the first 4 days after admission was correlated with Glasgow-Pittsburgh cerebral performance category 30 days after cardiac arrest, whereas the glucose was correlated with the outcomes until 3.5 days after admission^15^. A study suggested that a lactate/ pyruvate higher than 30 was correlated with low cerebral perfusion pressure (CPP), while it was not correlated with increased intra-cranial pressure (ICP) by investigating the data of 1873 cases^16^.

G/L_serum_ was found with a predictive value on 28-day mortality and 6-month GOSE, whereas it might not provide a valuable reference for the prediction of neurological complications. This result was achieved probably due to the following reasons. Most of the neurological complications in this study included delirium and agitation, which are mild neurological function impairments that are difficult to be assessed and differentiated by GCS score^17^. The above neurological complications were transient, and most of the patients could recover during the 24 h of ICU stay, so the GCS scores after 24 h of ICU stay might not show the above complications. As a result, the G/L_serum_ may not provide a valuable reference for the prediction of neurological complications.

As mentioned above, the elevated serum lactate was considered as a reflection of the elevation of intra-cerebral lactate concentration, the neurological function impairment in this study was associated with cerebral metabolism factor because patients with primary brain injury and secondary brain injury (unstable circulatory status) had been excluded before analysis. Lactate is an important cerebral metabolism substrate, it had been confirmed that intra-cerebral lactate and glucose will change their similar concentration and turn out to be a state of hypo-glucose and hyper-lactate concentration when the brain energy consumption balance is broken, which is termed acute metabolism crisis (ACMC)^18^. ACMC can arise from the primary brain injury and can be recognized as the etiology of secondary brain injury, causing acute neurological function impairment, and affecting the long-term outcome. A study of 14 TBI patients with one year follow-up indicated ACMC caused atrophy of frontal and parietal lobes and different degrees of impairment of attention, executive ability, cognitive ability, and emotional disorders^19^. A study of TBI patients confirmed the correlation between the cerebrospinal fluid (CSF) glucose/lactate ratio and ACMC^20^.

Reviewing the results, the serum lactate after the surgery was higher than normal commonly and it was considered as a reflection of the elevation of intracranial lactate concentration, this phenomenon revealed that the ACMC existed commonly after the surgery, the transformation and mobilization of energy substrates at the cellular level increased with increasing of energy demand. The serum lactate of the ΔGCS_24h_ decreased patients had no recovered trend during the 24 h of ICU stay, and the serum lactate of the ΔGCS_24h_ non-decreased patients had a recovered trend (Fig. 4), the consistent hyper-lactatemia reflected the consistent ACMC, and the consistent ACMC caused the imbalance of the brain’s homeostasis and neurological function impairment. A previous study found cerebral metabolism decreased under sedation ^21^, if proper sedation and analgesia can be implemented after neurosurgery, ACMC will probably not occur and neurological impairment would be reduced.

## Limitations

No comparison analysis was conducted between serum lactate and cerebral extra-cellular lactate concentration due to the deficiency of data monitored by CMD. Data of this study was from a single center, further study is needed by multiple centers studies, especially the accurate G/L_serum_ ratio cut-off value.

## Conclusions

Dynamic serum lactate monitoring and the G/L_serum_ at ICU admission can predict neurological function impairment after neurosurgery which might be attributed to ACMC.

## Data Availability

The datasets generated during and analyzed during the current study are available in the “SYNAPSE” repository, https://www.synapse.org/#!Synapse:syn29967387/files/. Contact the first author Dandan Liu to get the data. We are consent to publication if this manuscript is accepted.
